# ASSESSING THE SCALABILITY OF A COMMUNITY PROGRAM FOR OLDER ADULTS WITH DIABETES: BRIDGING THE RESEARCH–PRACTICE GAP

**DOI:** 10.1093/geroni/igad104.2961

**Published:** 2023-12-21

**Authors:** Melissa Northwood, Rebecca Ganann, Kathryn Fisher, Maureen Markle-Reid, Marie-Lee Yous

**Affiliations:** McMaster University, Hamilton, Ontario, Canada; McMaster University, Hamilton, Ontario, Canada; McMaster University, Hamilton, Ontario, Canada; McMaster University, Hamilton, Ontario, Canada; McMaster University, Hamilton, Ontario, Canada

## Abstract

Implementing person-centred, cost-effective, and comprehensive self-management programs for older adults with multimorbidity and their caregivers in the health and social care system is challenging. Innovations that are effective under controlled research conditions often fail to produce similar results when implemented in real world settings to reach larger populations of older adults. Scalability assessment is a promising methodology to reduce this research-practice gap. This study aims to examine the scalability of a self-management intervention for older adults with diabetes and multimorbidity and their caregivers in two Canadian provinces. Provincial working groups, including patient partners, participated in the scalability assessment. The Intervention Scalability Assessment Tool (ISAT) guided data collection and analysis. Multiple methods were used to collect data, including an environmental scan, document review, and interviews with key informants. Provincial workshops were held to review scalability results, determine the program’s scale-up readiness, and identify strategies to enhance scalability. Patient partners, health and social care providers and leaders, provincial decision-makers, and researchers gave high ratings to the readiness of the intervention and its alignment with practice and strategic policy initiatives. The evidence of effectiveness, delivery setting and workforce, and sustainability domains were rated lower. Participants recommended: 1) focusing on high-risk patients, which would be cost effective and likely to demonstrate impact and 2) piloting targeted sites to embed the program within existing health care settings and infrastructure to gather more evidence on program effectiveness and implementation. Collaborative and structured scalability assessments are critical to mobilize innovative programs into health and social care practice.

